# Association of Surgical Complications With Resource Utilization in Adult Congenital Cardiac Surgery

**DOI:** 10.1016/j.atssr.2023.03.018

**Published:** 2023-04-05

**Authors:** Catherine G. Williamson, Shayan Ebrahimian, Shineui Kim, Amulya Vadlakonda, Peyman Benharash

**Affiliations:** 1 Cardiovascular Outcomes Research Laboratories (CORELAB), David Geffen School of Medicine at UCLA, Los Angeles, California; 2 Department of Cardiac Surgery, David Geffen School of Medicine at UCLA, Los Angeles California

## Abstract

**Background:**

As patients with congenital heart disease are increasingly surviving well into adulthood, the morbidity, mortality, and resource utilization of adult congenital cardiac operations are of increasing interest. Therefore, we evaluated factors associated with perioperative morbidity and outcomes in adults undergoing congenital operations.

**Methods:**

The Nationwide Readmissions Database was tabulated for all adults (≥18 years old) with congenital heart disease between 2010 and 2017. Congenital operations were identified by previously published *International Classification of Diseases, Ninth Revision* and *Tenth Revision* codes. Complications were selected on the basis of The Society of Thoracic Surgeons short list of complications. Multivariable regression models were used to assess adjusted odds ratios (AORs) and β coefficients for select clinical outcomes.

**Results:**

Of 52,360 adults identified who underwent congenital cardiac operations, 14,123 (27%) suffered a complication. The presence of a complication increased the odds of index death (AOR, 11.46; 95% CI, 8.58-15.31), nonhome discharge (AOR, 2.09; 95% CI, 1.91-2.29), 30-day readmission (AOR, 2.12; 95% CI, 1.88-2.39), 90-day readmission (AOR, 2.17; 95% CI, 1.94-2.43), costs (β, +$37,000; 95% CI, $34,000-$40,000), and hospital duration (β, +7.86 days; 95% CI, 7.3-8.4).

**Conclusions:**

Perioperative complications portend in-hospital death, resource use, and readmissions in adults undergoing congenital heart operations. As complications are present in 27% of this population, identification of risk stratification and complication-reducing strategies may improve patient morbidity, mortality, and resource utilization.

**In Short**

▪ Surgical complications of adult congenital heart disease occurred in >25% of this population of patients.
▪ Perioperative morbidity in patients with adult congenital heart disease increases risk of in-hospital death, urgent readmissions, and discharge to secondary care facilities.
▪ Postoperative complications are responsible for an increase of more than $37,000 in hospitalization cost and 8-day increase in length of stay per patient.

As the US health care system continues to prioritize value-based care, the emphasis on cost reduction remains pertinent, especially within the surgical sector. A potential avenue for increasing value-driven care is through complication reduction, a known driver of hospitalization costs and increased hospitalization duration.^1^ This effect is particularly well documented in adult cardiac operations, with complications noted to increase length of stay and hospitalization costs after coronary artery bypass grafting by almost 50%.^2^ Yet, advances in surgical management of congenital heart disease have led to a new adult cardiac population in recent years. These improvements have enabled newborns with previously fatal conditions to survive well into adulthood, now representing 20% of cardiac surgical patients.^3^

Although several studies have previously shown that the experience and expertise of the health care team affect outcomes for adult patients with congenital heart disease, a thorough analysis of factors associated with adult congenital heart disease (ACHD) morbidity and its impact on resource utilization is lacking in the current literature. Therefore, this study used a nationally representative cohort to analyze factors associated with perioperative morbidity and resource utilization in adults with congenital heart disease.

## Material and Methods

The Nationwide Readmissions Database (NRD) was used to identify all adults (≥18 years) with congenital heart disease between 2010 and 2017. As the largest all-payer inpatient and readmission database in the United States, the NRD provides an accurate measurement of 58% of all national hospitalizations.

ACHD operations were identified by previously published *International Classification of Diseases, Ninth Revision* and *Tenth Revision* codes.^4^ These included operative revision for diagnoses such as ventricular or atrial septal defect, tetralogy of Fallot, transposition of the great vessels, hypoplastic left heart syndrome, truncus arteriosus, coarctation of aorta, and total anomalous pulmonary venous connection. Operative complexity was determined by The Society of Thoracic Surgeons–European Association for Cardio-Thoracic Surgery (STAT) scoring system.^5^

Using the NRD data dictionary, prepopulated variables including insurance status, median household income, age, and in-hospital death were defined. The Elixhauser Comorbidity Index, a previously validated score composed of 30 conditions, was used to quantify the burden of comorbidities for each patient. Patients were considered to have complications as defined by The Society of Thoracic Surgeons short list, which includes cardiac, intraoperative, mechanical circulatory support requirement, pulmonary, infectious, renal, and neurologic categories.^6^ The primary outcome of interest was resource utilization secondary to complications. Hospitalization costs were calculated by hospital-based cost-to-charge ratios, which were subsequently normalized to the 2017 Personal Health Care Index. Cumulative costs were calculated as the summation of index hospitalization costs and readmission costs.

Categorical variables are reported as frequencies (percentage), and nonnormally distributed continuous factors are reported as medians with interquartile ranges. Multivariable regression models adjusting for demographic and clinical characteristics were used to evaluate adjusted outcomes. The risk-adjusted association of covariates with specified outcomes is reported as adjusted odds ratios (AORs) for dichotomous outcomes and β coefficients for continuous variables, both with 95% CI. Statistical significance was set at α = .05. All analyses were preformed with Stata 16.1 (StataCorp LLC). This study was deemed exempt from full review by the institutional review board at the University of California, Los Angeles (IRB:17-001112, approved July 26, 2017).

## Results

### Cohort Demographics

**Figure 1:**
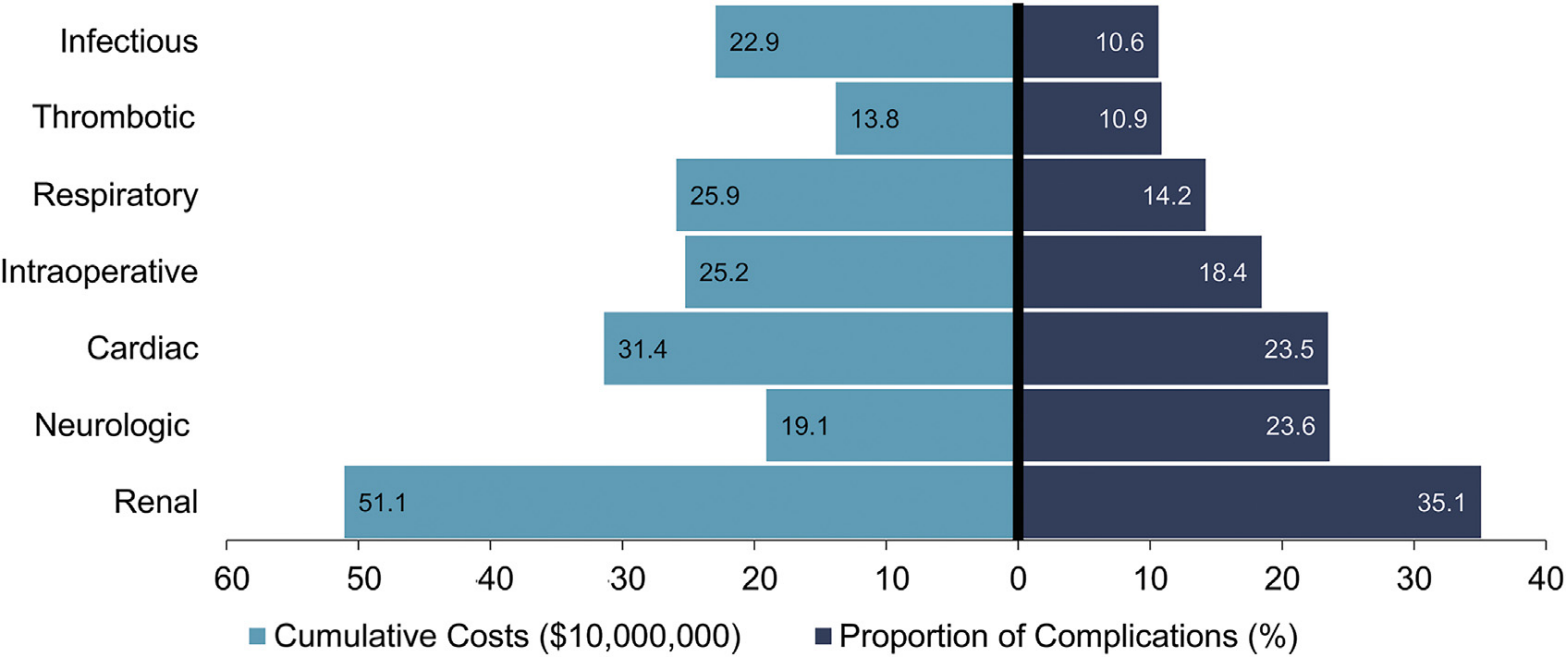
Numeric distribution and cumulative costs per complication type in the complicated cohort.

Of an estimated 52,360 adults identified who underwent congenital cardiac operations during the study period, 14,123 (27%) suffered a perioperative complication. Complication categories are summarized in Figure 1. Compared with those without a complication, this cohort was on average older (58.0 years vs 54.7 years; *P* < .001), more frequently male (57.9% vs 49.2%), and insured by government providers (Medicare, 46.2% vs 34.8% [*P* < .001]; Medicaid, 11.3% vs 10.3% [*P* < .001]; Table 1). This cohort also had a higher burden of comorbidities as measured by the Elixhauser Comorbidity Index (4.21 vs 3.12 points; *P* < .001) and underwent higher risk procedures as measured by the STAT score (1.21 vs 1.13; *P* < .001). In addition, patients with complications had higher rates of single-ventricle disease (0.8% vs 0.4%; *P* = .007) as well as all comorbidity groups except for chronic obstructive pulmonary disease (24.2% vs 24.6%; *P* = .69), hypertension (44.1% vs 47.8%; *P* = .001), obesity (10.3% vs 11.9%; *P* = .009), pulmonary hypertension (15.3% vs 15.6%; *P* < .001), and tobacco use (8.7% vs 8.9%; *P* = .67; Table 1). Complicated patients were less frequently treated at high-volume centers (74.3% vs 76.7%; *P* = .02), yet received care at similar proportions of teaching hospitals (81.6% vs 81.5%; *P* = .65) as well as centers in large metropolitan areas (67.6% vs 69.9%; *P* = .06) compared with those without a complication.

**Table 1:** Baseline Characteristics by Complication Status

Characteristic	Uncomplicated	Complicated	*P* Value
	(n = 38,237)	(n = 14,123)	
Demographics			
Age, y	54.7	58.0	<.001
Male	49.2	57.9	<.001
Elixhauser Comorbidity Index, points	3.12	4.21	<.001
Elective admission	73.1	43.5	<.001
Insurance			<.001
Medicare	34.8	46.2	
Medicaid	10.3	11.3	
Private	48.7	35.2	
Uninsured	2.3	3.7	
Income quartile			<.001
0-24th percentile	21.1	25.3	
25th-49th percentile	24.4	24.8	
50th-74th percentile	25.3	25.7	
75th-99th percentile	29.2	24.2	
Comorbidities			
STAT score, points	1.13	1.21	<.001
Single-ventricle disease	0.4	0.8	.007
Alcohol abuse	1.6	2.6	.001
Arrhythmias	40.1	51.7	<.001
Coronary artery disease	29.5	32.9	.001
Congestive heart failure	22.5	36.7	<.001
Coagulopathy	13.9	21.8	<.001
COPD	24.6	24.2	.69
Diabetes mellitus	15.0	16.4	.02
Hypertension	47.8	44.1	.001
Obesity	11.9	10.3	.009
Pulmonary hypertension	15.6	15.3	.69
Liver disease	1.6	5.7	<.001
Renal failure	5.6	13.6	<.001
Smoker	8.9	8.7	.67
Hospital factors			
Center volume			.02
Low	6.8	7.2	
Medium	16.5	18.6	
High	76.7	74.3	
Hospital urban-rural designation			.06
Large metropolitan	69.9	67.6	
Small metropolitan	29.1	31.2	
Micropolitan	1.0	1.1	
Teaching center	81.5	81.6	.65

Categorical variables are presented as percentage.

COPD, chronic obstructive pulmonary disease; STAT, The Society of Thoracic Surgeons–European Association for Cardio-Thoracic Surgery.

### Unadjusted Analysis

On bivariate comparison, patients with complications during the index admission suffered higher rates of in-hospital death (8.1% vs 0.5%; *P* = .01), nonhome discharge (58.7% vs 35.5%; *P* < .001), and unplanned readmissions within 30 days (21.9% vs 10.9%; *P* < .001) and 90 days (29.2% vs 14.8%; *P* < .001) after discharge (Table 2). Complicated patients also accrued higher index hospitalization costs ($82,700 vs $38,800; *P* < .001), costs on readmissions ($59,991 vs $22,352; *P* < .001), and cumulative costs ($102,111 vs $45,370; *P* < .001). Furthermore, the complicated cohort experienced a longer length of stay at index hospitalization (15.9 days vs 6.4 days; *P* < .001; Table 2). As noted in Figure 1, infectious causes of complications disproportionally contributed to total costs, representing 10.6% of complicated patients yet accounting for $229 million in total expenditures (Figure 1).

**Table 2:** Unadjusted Outcomes by Complication Status

Outcome	Uncomplicated	Complicated	*P* Value
	(n = 38,237)	(n = 14,123)	
In-hospital death	0.5	8.1	.01
Cost, $	38,800	82,700	.98
Hospital duration, d	6.4	15.9	.34
Nonhome discharge	35.5	58.7	.49
30-day readmission	10.9	21.9	.19
90-day readmission	14.8	29.2	.96
No. of times readmitted	5.25	7.98	.0001
Readmission costs at 30 days, $	22,354	59,991	<.001
Cumulative costs, $	45,370	102,111	<.001
Care fragmentation on readmission	21.2	15.0	.28

Categorical variables are presented as percentage.

### Adjusted Analysis

**Figure 2:**
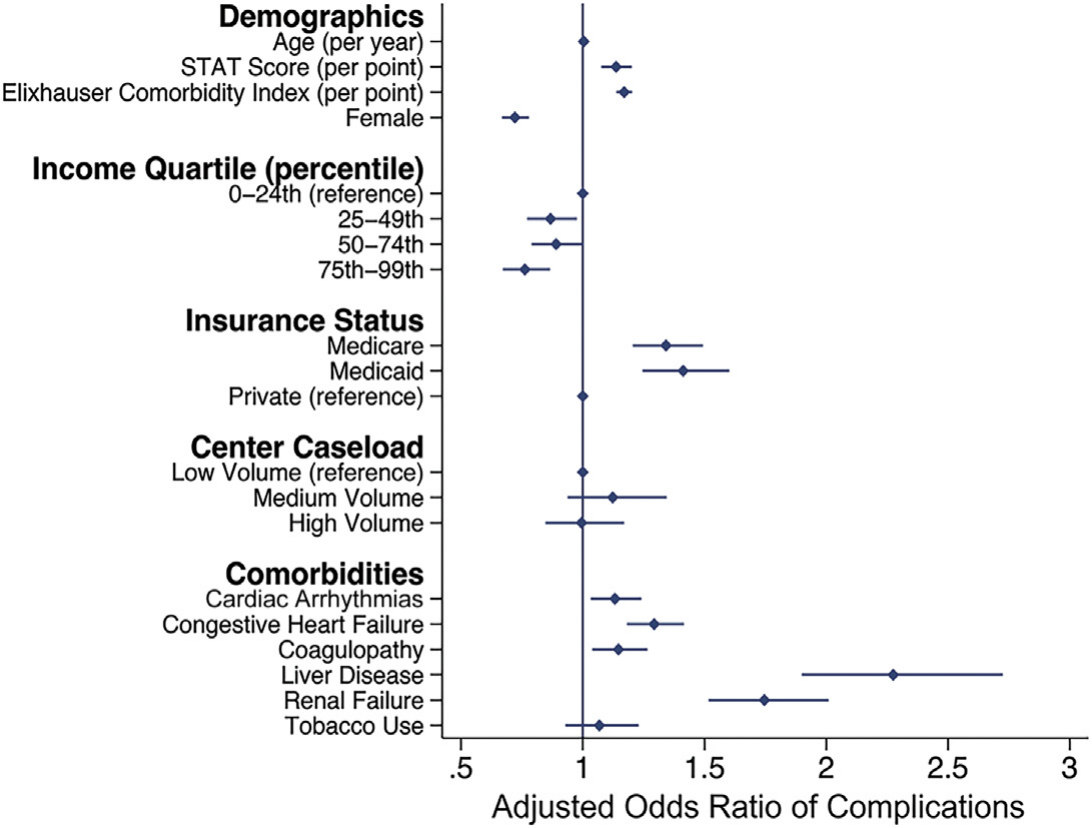
Factors associated with experiencing a complication. (STAT, The Society of Thoracic Surgeons–European Association for Cardio-Thoracic Surgery.)

After adjusted multivariable analysis, factors associated with increased odds of perioperative complications included STAT score, Elixhauser Comorbidity Index, governmental insurance coverage, male sex, and income quartile (Figure 2). The presence of a complication increased the odds of index death (AOR, 11.46; 95% CI, 8.58-15.31), nonhome discharge (AOR, 2.09; 95% CI, 1.91-2.29), 30-day readmission (AOR, 2.12; 95% CI, 1.88-2.39), 90-day readmission (AOR, 2.17; 95% CI, 1.94-2.43), costs (β, +$37,000; 95% CI, $34,000-$40,000), and hospital duration (β: +7.86 days; 95% CI, 7.3-8.4).

## Comment

Defining the impact of perioperative complications in the ACHD cohort is crucial to improving value-based, high-quality surgical care for this population.^1^ In our study, we found that perioperative complications portend greater burden of in-hospital death, resource use, and readmissions in adults undergoing congenital heart operations. Notably, complications increased hospitalization costs by almost $40,000 per patient after adjustment, with a concomitant 8-day increase in length of stay. Yet, a complication occurred in >25% of this population of patients. The significant financial and clinical burden of complications in this high-risk cohort requires further discussion.

Composing more than a quarter of the study population, patients in the complication subgroup cost approximately twice that of other patients and experienced a >2-fold increase in length of stay. These findings imply that during the study period, approximately $522 million was attributable to perioperative morbidity during the index admission. In the current era of growing health care expenditures, hospitals and policymakers are increasingly focused on cost reduction, without sacrificing patient outcomes. Complication reduction may allow such improvements while simultaneously improving patient outcomes. In a population of pediatric congenital heart surgery patients, Pasquali and colleagues^7^ estimated a 10% decrease in major complications and 1- to 3-day decrease in length of stay to reduce costs by more than $20 million in just 5 years. Several potential opportunities have been proposed to reduce perioperative morbidity, including expedited intensive care unit downgrades, extubation, and mobilization. Moreover, hospital collaboratives, such as the Pediatric Cardiac Critical Care Consortium, have shown significant promise for improving pediatric congenital cardiac outcomes through quality improvement identification as well as implementation.^8^ This collaborative may be worth replicating in the ACHD population to share information between centers and to pursue value-based solutions.

In this study, those with perioperative morbidity experienced 11-fold increase in odds of in-hospital death, 2-fold increase in odds of discharge to a secondary care facility, and 2-fold increase in odds of unplanned readmission within 30 days compared with those without. These findings indicate the immense clinical burden of complications on patients and their families. Whereas complications increased resource utilization in the index hospitalization, the financial burden of such morbidities may prove to be even greater because of the costs of posthospitalization readmissions and requirement of secondary care facilities. For example, readmissions for both medical and surgical indications were estimated to cost approximately $17.4 billion per year in total across a cohort of Medicare beneficiaries.^9^ Moreover, secondary care facilities, although less costly than inpatient hospitalizations, further increase the total cost burden attributable to perioperative morbidity.^10^ In sum, complications affect not only outcomes during the index hospitalization but also clinical and financial well-being after hospital discharge.

As with many administrative databases, the NRD is limited by reliance on hospital billing preferences and manual coding, which may affect the study data. Furthermore, the NRD is unable to provide specific clinical information, including illness severity, laboratory values, and imaging. Moreover, patients with ACHD span a wide range of surgical difficulty and clinical severity as measured by the STAT score, a validated numerical indicator of surgical difficulty for each patient.^5^ Yet, the full spectrum of ACHD cannot be fully captured without granular clinical data. Despite these limitations, we used the largest all-payer readmissions database to evaluate the resource utilization of morbidity in a high-risk adult cohort with ACHD.

In conclusion, perioperative morbidity in the ACHD population increases risk of in-hospital death, urgent readmissions, and discharge to secondary care facilities. Notably, these complications are responsible for more than $37,000 increase in hospitalization cost and 8-day increase in length of stay per patient. As complications are frequent in this population, identification of risk stratification and complication-reducing strategies may improve patient morbidity, mortality, and resource utilization.
